# Advancing Neonatal Screening for Pyridoxine-Dependent Epilepsy-ALDH7A1 Through Combined Analysis of 2-OPP, 6-Oxo-Pipecolate and Pipecolate in a Butylated FIA-MS/MS Workflow

**DOI:** 10.3390/ijns11030059

**Published:** 2025-07-30

**Authors:** Mylène Donge, Sandrine Marie, Amandine Pochet, Lionel Marcelis, Geraldine Luis, François Boemer, Clément Prouteau, Samir Mesli, Matthias Cuykx, Thao Nguyen-Khoa, David Guénet, Aurélie Empain, Magalie Barth, Benjamin Dauriat, Cécile Laroche-Raynaud, Corinne De Laet, Patrick Verloo, An I. Jonckheere, Manuel Schiff, Marie-Cécile Nassogne, Joseph P. Dewulf

**Affiliations:** 1 Department of Pediatric Neurology, Kannerklinik Centre Hospitalier du Luxembourg, L-1210 Luxembourg, Luxembourg; 2 Biochemical Genetics and Newborn Screening Laboratory, Department of Laboratory Medicine, Cliniques Universitaires Saint-Luc, UCLouvain, B-1200 Brussels, Belgium; 3 Louvain Centre for Toxicology and Applied Pharmacology, Institut de Recherche Experimentale et Clinique, UCLouvain, B-1200 Brussels, Belgium; 4 Laboratory of Paediatric Research and Newborn Screening, Université Libre de Bruxelles, B-1020 Brussels, Belgium; 5 Biochemical Genetics and Newborn Screening Laboratory, CHU Liege, B-4000 Liege, Belgium; 6 Department of Medical Genetics, Angers University Hospital, F-49000 Angers, France; 7 Department of Biochemistry, Centre Hospitalier Universitaire de Bordeaux, F-33404 Bordeaux, France; 8 Clinical Chemistry and Newborn Screening Center, UZ Antwerpen, B-2650 Antwerpen, Belgium; 9 Laboratoire du Centre Régional de Dépistage Néonatal de l’Ile de France, Hôpital Necker-Enfants Malades, AP-HP Centre Université Paris Cité, 161, rue de Sèvres, F-75015 Paris, France; 10 INSERM U1151, Institut Necker Enfants Malades, F-75015 Paris, France; 11 Laboratory of Biochemistry, Normandie Université, UNICAEN, CHU of Caen Normandie, F-14000 Caen, France; 12 Nutrition and Metabolic Clinic, Brussels University Hospital, Academic Children Hospital Queen Fabiola, Université Libre de Bruxelles, B-1020 Brussels, Belgium; 13 Medical Genetics and Cytogenetics Department, Limoges Universitary Hospital, F-87000 Limoges, France; 14 Centre de Compétence des Maladies Héréditaires du Métabolisme, Centre Hospitalier Universitaire de Limoges, F-87000 Limoges, France; 15 Department of Pediatric Neurology, Center for Inherited Metabolic Disorders, University Hospital Ghent, B-9000 Ghent, Belgium; 16 Centre for Metabolic Diseases, University Hospital Antwerp, University of Antwerp, B-2650 Antwerpen, Belgium; 17 Reference Center for Inborn Errors of Metabolism, Necker University Hospital, AP-HP Centre Université Paris Cité, Filière G2M, F-75015 Paris, France; 18 INSERM UMRS_1163, Institut Imagine, F-75015 Paris, France; 19 Department of Pediatric Neurology, Cliniques Universitaires Saint-Luc, UCLouvain, B-1200 Brussels, Belgium; 20 Institut des Maladies Rares, Cliniques Universitaires Saint-Luc, UCLouvain, B-1200 Brussels, Belgium

**Keywords:** pyridoxine-dependent epilepsy, ALDH7A1, 2-OPP, 6-oxo-pipecolate, pipecolate, FIA-MS/MS, newborn screening, dried blood spots, 2S,6S-/2S,6R-oxopropylpiperidine-2-carboxylate

## Abstract

Pyridoxine-dependent epilepsy (PDE) represents a group of rare developmental and epileptic encephalopathies. The most common PDE is caused by biallelic pathogenic variants in *ALDH7A1* (PDE-ALDH7A1; OMIM #266100), which encodes α-aminoadipate semialdehyde (α-AASA) dehydrogenase, a key enzyme in lysine catabolism. Affected individuals present with seizures unresponsive to conventional anticonvulsant medications but responsive to high-dose of pyridoxine (vitamin B6). Adjunctive lysine restriction and arginine supplementation have also shown potential in improving neurodevelopmental outcomes. Given the significant benefit of early intervention, PDE-ALDH7A1 is a strong candidate for newborn screening (NBS). However, traditional biomarkers are biochemically unstable at room temperature (α-AASA and piperideine-6-carboxylate) or lack sufficient specificity (pipecolate), limiting their utility for biomarker-based NBS. The recent identification of two novel and stable biomarkers, 2S,6S-/2S,6R-oxopropylpiperidine-2-carboxylate (2-OPP) and 6-oxo-pipecolate (oxo-PIP), offers renewed potential for biochemical NBS. We evaluated the feasibility of incorporating 2-OPP, oxo-PIP, and pipecolate into routine butylated FIA-MS/MS workflows used for biochemical NBS. A total of 9402 dried blood spots (DBS), including nine confirmed PDE-ALDH7A1 patients and 9393 anonymized controls were analyzed using a single multiplex assay. 2-OPP emerged as the most sensitive biomarker, identifying all PDE-ALDH7A1 patients with 100% sensitivity and a positive predictive value (PPV) of 18.4% using a threshold above the 99.5th percentile. Combining elevated 2-OPP (above the 99.5th percentile) with either pipecolate or oxo-PIP (above the 85.0th percentile) as secondary marker detected within the same multiplex FIA-MS/MS assay further improved the PPVs to 60% and 45%, respectively, while maintaining compatibility with butanol-derivatized method. Notably, increasing the 2-OPP threshold above the 99.89th percentile, in combination with either pipecolate or oxo-PIP above the 85.0th percentile resulted in both 100% sensitivity and 100% PPV. This study supports the strong potential of 2-OPP-based neonatal screening for PDE-ALDH7A1 within existing NBS infrastructures. The ability to multiplex 2-OPP, pipecolate and oxo-PIP within a single assay offers a robust, practical, high-throughput and cost-effective approach. These results support the inclusion of PDE-ALDH7A1 in existing biochemical NBS panels. Further prospective studies in larger cohorts are needed to refine cutoffs and confirm clinical performance.

## 1. Introduction

Pyridoxine-dependent epilepsy related to ALDH7A1 deficiency (PDE-ALDH7A1; OMIM #266100) is a rare developmental epileptic encephalopathy characterized by refractory seizures that respond to a high dose of pyridoxine (vitamin B6) [1]. ALDH7A1 is an enzyme responsible for the oxidation of alpha-aminoadipate semialdehyde (α-AASA) to α-aminoadipate in the lysine degradation pathway. A congenital deficiency of this enzyme leads to the accumulation of α-AASA and piperideine-6-carboxylate (P6C) in body fluids [2,3,4], the latter resulting from spontaneous cyclization of α-AASA [5,6]. Biochemically, P6C reacts with pyridoxal-5′-phosphate cofactor (the active form of vitamin B6), leading to its inactivation. Pipecolate (PIP), another intermediate in the lysine pathway is also typically elevated in this metabolic disorder. However, it is a less specific biomarker for PDE-ALDH7A1, as elevated levels can also be observed in other metabolic conditions such as peroxisomal diseases, hyperlysinemia or liver damage [7,8,9,10,11]. Despite effective seizure control by pyridoxine supplementation, approximately 75% of patients experience global developmental delay and/or intellectual disability [12,13]. Recent therapeutic approaches aimed to reduce α-AASA and P6C accumulation by implementing lysine reduction strategies, including a lysine-restricted diet combined with arginine supplementation [14,15,16,17,18]. Combined with pyridoxine supplementation, these approaches have been shown to improve cognitive outcomes, especially if initiated within the first few months of life [13,19].

However, diagnosis of PDE-ALDH7A1 is often delayed [1,20] owing to the nonspecific nature of the symptoms, the wide variability in the age of clinical onset [21,22,23,24], and the lack of routine neonatal testing. Given the clear therapeutic benefit of early intervention, PDE-ALDH7A1 is considered a strong candidate for inclusion in newborn screening (NBS) programs [25,26,27]. Nonetheless, α-AASA and P6C are not ideal screening biomarkers in dried blood spots (DBS) because of their instability at room temperature [28,29].

Recently, new studies have identified 6-oxo-pipecolate (oxo-PIP) and 2S,6S-/2S,6R-oxopropylpiperidine-2-carboxylate (2-OPP) as novel, more stable biomarkers for PDE-ALDH7A1 diagnosis [27,30,31,32]. A limited but promising preliminary study demonstrated a significant increase in 2-OPP levels in neonatal DBS from two PDE-ALDH7A1 patients [30] compared to ten controls, using a non-derivatized flow injection-tandem mass spectrometry (FIA-MS/MS) assay, a method traditionally used in high-throughput NBS. During the preparation of this manuscript, two independent studies confirmed the suitability of 2-OPP and oxo-PIP measurements for PDE-ALDH7A1 screening in neonatal DBS, also using non-derivatized FIA-MS/MS assays and including two-tiered approaches [31,32].

In this study, we incorporated MS/MS specific biochemical transitions for PDE-ALDH7A1 biomarkers in our FIA-MS/MS newborn screening assay, utilizing butanol for derivatization, a method commonly used for measuring amino acid and acylcarnitine in routine NBS. We assessed butylated 2-OPP, oxo-PIP and PIP in 9393 fresh control neonatal DBS samples and measured these three biomarkers in nine neonatal leftover DBS samples from confirmed PDE-ALDH7A1 patients.

## 2. Materials and Methods

### 2.1. Patient Cohort and Control Samples

This study included patients with genetically confirmed PDE-ALDH7A1, enrolled through the multicenter academic BUSARD initiative (Blood spot and Urine metabolomics Screening Applied to Rare Diseases, ClinicalTrials.gov ID NCT06360913). Eligibility required biallelic pathogenic variants in *ALDH7A1* gene and the availability of a residual newborn DBS collected at birth. Informed consent was obtained for all and the BUSARD protocol was approved by the local ethics committee (study number: 2023/09AOU/346).

A total of 9393 anonymized DBS samples from newborns served as controls. These were selected from Belgian French-speaking NBS centers and had tested negative for all disorders in our standard NBS panel: congenital hypothyroidism, congenital adrenal hyperplasia, cystic fibrosis, spinal muscular atrophy, sickle cell disorders, biotinidase deficiency, galactosemia, phenylketonuria, tyrosinemia, homocystinuria, maple syrup, urine disease, methylmalonic acidemia, propionic acidemia, isovaleric acidemia, glutaric acidemia type I, MCAD deficiency, VLCAD deficiency, multiple acyl-CoA dehydrogenase deficiency, LCHAD deficiency, carnitine uptake deficiency, CPT1 deficiency, HMG-CoA lyase deficiency and acetoacetyl-CoA thiolase deficiency. Control samples were collected between 48 and 96 h of life, either from babies born after at least 36 weeks of gestational age (n = 9349) or premature infants with a gestational age of 26 to 36 weeks (n = 44).

### 2.2. Sample Preparation, FIA-MS/MS Analysis and Validation

A 1/8-inch (3.2 mm) punch corresponding to ~3.2 µL of blood from each DBS was transferred to a 96-well plate and extracted with 190 µL of the NBS extraction solution, containing internal standards of amino acids and acylcarnitines in methanol (Cambridge Isotope Laboratories, reference NSK-AB). The plate was covered with aluminum foil and allowed to sit at room temperature for 30 min. The eluate was subsequently transferred to a new 96-well plate and evaporated under nitrogen at 45 °C. The dry residue was then subjected to butylation by adding 50 µL of butylation reagent to each well. This reagent was prepared by mixing nine volumes of 1-butanol (Merck, Rahway, NJ, USA, reference 1.01990) with one volume of acetyl chloride (Merck, reference 00990). The plate was incubated at 60 °C for 15 min while covered with aluminum foil. Following complete evaporation under nitrogen, the dry residue was resuspended in 200 µL of acetonitrile/water (50:50), and the plate was shaken for 5 min at 600 rpm. Before injection, each sample was diluted 1:10 with 50% acetonitrile in a new plate.

A volume of 10 µL from each well was injected by FIA-MS/MS, using a Waters Xevo-TQ-S micro mass spectrometer equipped with an electrospray ionization (ESI) source operating in positive mode. The ESI parameters were set as follows: capillary voltage 3.0 kV, drying gas temperature at 300 °C and gas flow rate at 600 L/h. Analytical standards were purchased from Sigma/Merck (6-oxo-pipecolate: reference 36323, pipecolate: reference 2519) and from Synvenio B.V. (Nijmegen, The Netherlands) for 2S,6S-/2S,6R-oxopropylpiperidine-2-carboxylate/2-OPP (mix of diastereoisomers in ~1:1 ratio). Biochemical MS/MS transitions and collision energies used for butylated molecules were optimized after infusion of concentrated standard solutions: 2-OPP (*m/z* 242 > 82, 26 eV), oxo-PIP (*m/z* 200 > 98, 18 eV) and PIP (*m/z* 186 > 84, 18 eV). Concentrations were estimated based on the relative response to the internal standard ^13^C6-Phenylanine, which was added at a known concentration during the sample preparation for FIA-MS/MS neonatal screening. The measured values are therefore reported as relative concentrations. Linearity ranges, precision and reproducibility for the three analytes were established by spiking known concentrations into washed red blood cells and physiological saline solution as described previously [33]. Calibration curves demonstrated good linearity within the following ranges: 0.08–20 μmol/L for 2-OPP, 0.1–20 μmol/L for oxo-PIP and 2.2–450 μmol/L for PIP. We analyzed six replicates of three control levels within the same batch to assess intra-day precision and performed three separate runs to evaluate inter-day reproducibility (Table 1). All coefficients of variation (CVs) for intra-day and inter-day measurements were below 15% and 18%, respectively, indicating good precision and reproducibility. Injection of three blank samples following the highest control level showed no detectable carry-over.

We assessed the matrix effect by comparing the signal of standards in water or in an eluate of DBS made from washed red blood cells. Surprisingly, in the DBS from control washed red blood cells, a high basal signal of PIP was detected ranging from ~20 to ~60 µmol/L, while 2-OPP and oxo-PIP were nearly undetectable. These findings suggest that the relative concentrations of PIP in DBS are likely overestimated due to matrix effects, potentially caused by isobaric interference. Since both 5-oxoproline (either endogenous or formed from glutamine degradation during the butylation process [34]) and PIP share a similar molecular mass (*m/z* 186 after butylation), we investigated this possibility further. We spiked washed red blood cells with 200 µmol/L of PIP, 5-oxoproline or glutamine prior to preparing DBS samples, and assessed potential isobaric interference with PIP measurement under our method conditions (*m/z* 186 > 84, 18 eV).

The results confirmed partial isobaric interference from both 5-oxoproline and glutamine, corresponding to approximately 20% and 10% of the PIP signal, respectively, relative to the 100% signal observed after PIP spiking alone.

Damiano et al. [31] previously demonstrated that both 2-OPP and oxo-PIP exhibit good stability on DBS at room temperature, with CVs below 10% for up to 28 days. In our study, we evaluated the long-term stability of these three analytes in DBS samples from three patients after six months of storage at room temperature. All CVs were below 21%, indicating acceptable stability and no clear degradation for extended storage in DBS format.

### 2.3. Statistics

Performance indicators and percentile calculations were performed in Excel. Sensitivity and specificity were calculated along with 95% confidence intervals (CIs) using the Wilson/Brown method in GraphPad Prism version 9.5.1.

## 3. Results

### 3.1. Nine Genetically Confirmed PDE-ALDH7A1 Patients Were Recruited for This Study

Nine patients with genetically confirmed PDE-ALDH7A1 diagnosis were included in this study. Seven presented with neonatal seizures, while two were diagnosed at birth based on a positive family history. All patients received pyridoxine supplementation prior to genetic confirmation (Table 2). Neonatal DBS samples were collected within the first days of life and stored at room temperature for 9 months to 5 years prior to biochemical analysis for the study (Table 2).

### 3.2. 2-OPP Is a Highly Sensitive and Reasonably Specific Biomarker for PDE-ALDH7A1 Newborn Screening via Butanol-Derivatization FIA-MS/MS

**Figure 1:**
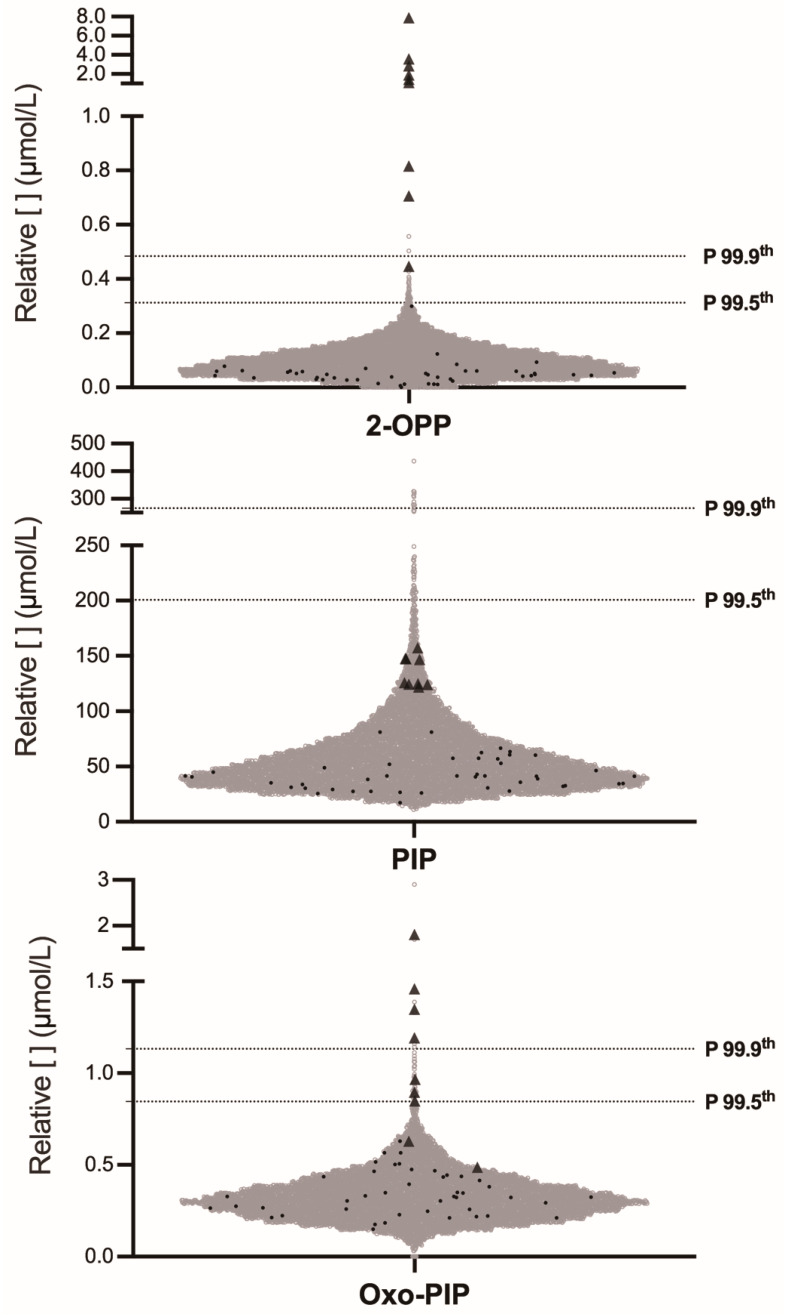
Distribution of PDE-ALDH7A1 biomarker levels in neonatal DBS using a butanol-derivatized FIA-MS/MS assay. PDE-ALDH7A1 Patients (black triangles, n = 9) and controls (empty grey disks, n = 9349; black disks corresponding to premature infants with a gestational age of 26 to 36 weeks, n = 44) are represented. Dashed lines represent the 99.5th and 99.9th percentiles for each molecule.

**Figure 2:**
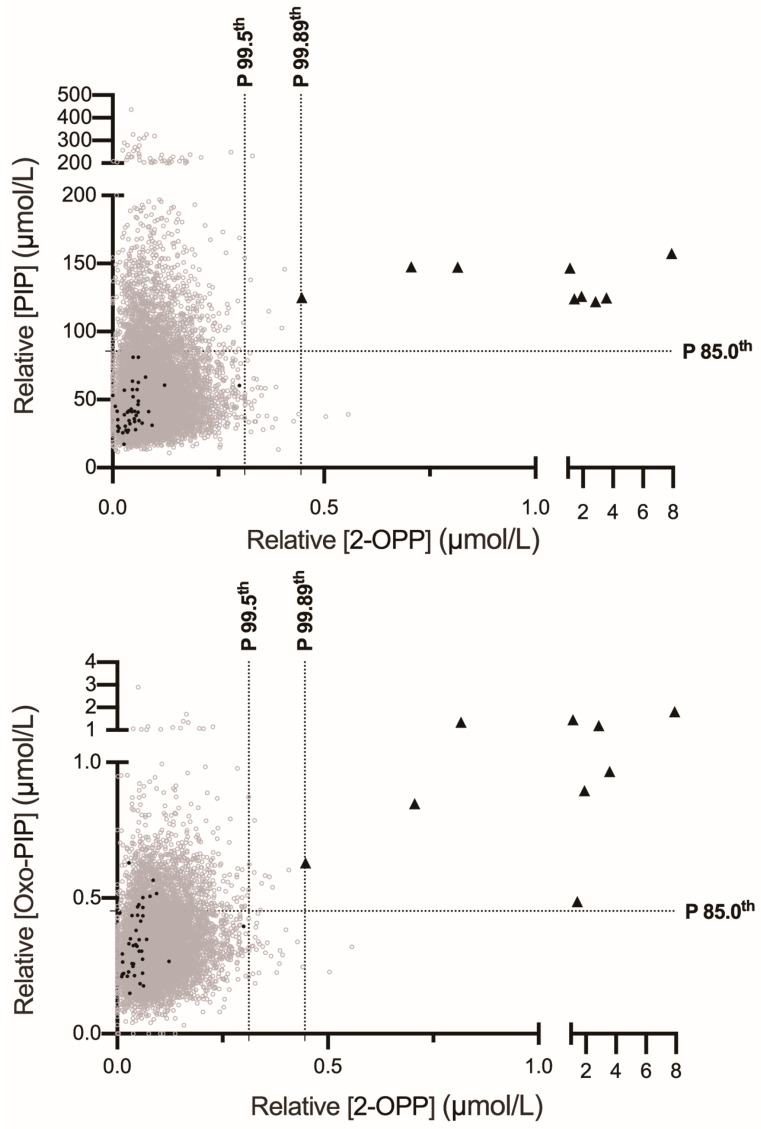
Combination of 2-OPP and additional PDE-ALDH7A1 biomarkers. PDE-ALDH7A1 patients (black triangles, n = 9) and controls (empty grey disks, n = 9349; black disks corresponding to premature infants with a gestational age of 26 to 36 weeks, n = 44) are represented. Vertical dashed lines represent the 99.5th and 99.89th percentiles for 2-OPP. Horizontal dashed lines represent the 85.0th percentiles for PIP and oxo-PIP.

The 2-OPP, 6-oxo-pipecolate (oxo-PIP) and pipecolate (PIP) were butylated prior to analysis by FIA-MS/MS using a multiplex method that simultaneously measured amino acids and acylcarnitines. The inclusion of specific MS/MS transitions for these three compounds within the existing FIA-MS/MS screening protocol represents an interesting approach for NBS extension, introducing minimal additional cost or complexity. Levels of 2-OPP in 8/9 PDE-ALDH7A1 patients were above the 99.9th percentile and all were above the 99.5th percentile (Figure 1), underscoring a high sensitivity (Figure 2). Among the 9393 control samples, 40 exceeded the 99.5th percentile, indicating reasonably good specificity as well, though there remains room for improvement. The positive predictive value (PPV) for 2-OPP at the 99.5th percentile was calculated at 18.4% (Table 3).

In contrast, oxo-PIP showed suboptimal performance as a primary biomarker for PDE-ALDH7A1 NBS. Among the nine confirmed cases, only four patients had oxo-PIP levels above the 99.9th percentile, and 7/9 exceeded the 99.5th percentile (Figure 1), indicating lower sensitivity compared to 2-OPP. Specificity was similar to 2-OPP, with 40 false positives above the 99.5th percentile and six above the 99.9th percentile (compared to only two for 2-OPP).

As expected, PIP was neither a sensitive nor a specific biomarker for primary screening of PDE-ALDH7A1. Even considering that the reported relative concentrations in DBS are likely overestimated (see Methods and Discussion), none of the nine confirmed patients exhibited PIP levels exceeding the 99.5th percentile (Figure 1).

Finally, we assessed the levels of our three biomarkers in 44 residual DBS samples from premature infants (gestational age 26 to 36 weeks, black disks in Figure 1) to evaluate whether prematurity could potentially lead to false-positive results. Although the sample size was limited, all tested DBS values were below the 99.5th percentile for each of the three biomarkers. The mean ± 2SD values for this population were as follows: 0.05 ± 0.09 µmol/L for 2-OPP, 0.34 ± 0.23 µmol/L for oxo-PIP, and 42.8 ± 29.3 µmol/L for PIP.

### 3.3. Combining 2-OPP with Pipecolate or 6-Oxo-Pipecolate Enhances Specificity and Positive Predictive Value in Multiplex FIA-MS/MS Screening for PDE-ALDH7A1

Given that all three butylated biomarkers (2-OPP, oxo-PIP, and PIP) were included in our multiplex FIA-MS/MS method, we evaluated whether the false-positive results observed with 2-OPP screening alone could be further refined using the additional measured markers. To explore this hypothesis, individual values for 2-OPP and either PIP or oxo-PIP were plotted in two-dimensional distribution graphs for both patients and controls (Figure 2). The application of this two-factor algorithm, the combination of 2-OPP with either PIP or oxo-PIP at cutoff-percentiles of 99.89% for 2-OPP and 85.0% for PIP or oxo-PIP, was found to be superior to the 2-OPP-alone screening strategy. This combination increased the PPV to 60.0% (with PIP) or 45.0% (with oxo-PIP) (Table 3). The combination of 2-OPP with PIP performed slightly better than the 2-OPP with oxo-PIP strategy, yielding 6 or 11 false positives, respectively, among the 9393 negative controls, while both approaches maintained 100% sensitivity (Figure 2 and Table 3). Additionally, we calculated the PPVs using combinations of 2-OPP at the 99.89th percentile with either PIP or oxo-PIP at the 85th percentile. Both combinations achieved maximal performances with 100% sensitivity and 100% PPV (Figure 2 and Table 3).

## 4. Discussion

This study of nine PDE-ALDH7A1 patients and 9393 negative controls supports 2-OPP as a reliable and high-performing primary biomarker for the biochemical screening of pyridoxine-dependent epilepsy due to ALDH7A1 deficiency.

Our findings demonstrate that 2-OPP can be effectively incorporated into a butylated FIA-MS/MS workflow, allowing for a high-throughput, cost-effective, and efficient neonatal screening of PDE-ALDH7A1. This method is readily adaptable within existing NBS programs [28,30], requiring only the purchase of MS-grade standards for the biomarkers. This addition represents minimal extra cost within an established workflow. However, costs associated with method validation and, ideally, compliance with ISO 15189 accreditation requirements also need to be considered and estimated. The fees and requirements related to accreditation and audits may vary between countries.

In all nine confirmed patients recruited in this study (and tested within 5 years of collection), 2-OPP levels exceeded the 99.5th percentile when compared to a control cohort of 9393 samples. This demonstrates excellent sensitivity while the calculated PPV of 18.4% remains within a reasonable range. Our data strongly suggest that 2-OPP is a leading biomarker for PDE-ALDH7A1 NBS. Additionally, when combined with secondary biomarkers such as PIP or oxo-PIP, it offers even higher positive predictive value without requiring second-tier testing, particularly in workflows using butanol-derivatized FIA-MS/MS. Importantly, when applying the screening strategy that uses elevated 2-OPP as the primary biomarker and PIP as a secondary marker, isolated increases in PIP without a corresponding elevation in 2-OPP should not be reported. This is due to the high risk of false-positive results. Isolated PIP elevations may occur in other metabolic conditions, particularly peroxisomal disorders (see Section 1), or result from isobaric interferences such as 5-oxoprolinemia or hyperglutaminemia (see Section 2.2). These conditions can be associated with inborn errors of metabolism, including 5-oxoprolinase deficiency, glutathione synthetase deficiency [35], or metabolic causes of hyperglutaminemia, as well as secondary causes.

Moreover, elevated PIP concentrations were observed in Figure 1 among several newborns with no available clinical information other than gestational age over 36 weeks and a negative screen for all 23 conditions currently included in the French-speaking Belgian NBS program. This finding suggests that unexplained isolated PIP elevations may also occur in other, as-yet unidentified conditions.

Our results align with two feasibility studies published during the preparation of this manuscript [31,32], which instead proposed a two-tiered strategy. These studies utilized an underivatized FIA-MS/MS assay for initial biochemical screening, followed by LC-MS/MS quantification of oxo-PIP as a second-tier test. The numbers of PDE-ALDH7A1 residual neonatal DBS analyzed were as follows: Pauly et al. (n = 8; among these, five samples tested 7–33 years post-collection), Damiano et al. (n = 2; one sample tested six years post-collection). Pauly et al. identified increased oxo-PIP in eight samples, seven of which also showed elevated 2-OPP. The single discordant sample (normal for 2-OPP), was collected three decades earlier, suggesting a possible biomarker degradation. Similarly, Damiano et al. analyzed two residual neonatal DBS samples and found 2-OPP to be more sensitive than oxo-PIP, consistent with our findings testing nine independent residual neonatal DBS samples, all collected a maximum of five years before analysis. Notably, these two studies did not use direct multiplexing of oxo-PIP in underivatized FIA-MS/MS because the detection of this compound is hindered by isobaric interferences in control DBS samples [30]. In contrast, our derivatized butanol-based FIA-MS/MS method successfully resolved the challenge of detecting oxo-PIP, potentially eliminating the need for a second-tier LC-MS/MS assay. Additionally, we observed that elevated PIP, when combined with 2-OPP in primary screening, enhances specificity as well. Therefore, we propose evaluating whether pipecolate could be multiplexed as a secondary biomarker in underivatized FIA-MS/MS screening workflows. This approach may prevent the need for second-tier LC-MS/MS testing of oxo-PIP in biochemical newborn screening protocols using underivatized FIA-MS/MS.

As described in the Section 2, the concentrations reported in our screening assay are based on signal intensity relative to an internal standard and are therefore expressed as relative concentrations, a strategy previously adopted by others [32]. We acknowledge that using stable isotope-labeled standards for each biomarker would improve accuracy. Additionally, PIP-relative concentrations are likely overestimated in our assay, as evidenced by a consistently and variable basal signal detected in control washed red blood cells, possibly due to interference from isobaric compounds. Previously published plasma PIP concentrations in affected PDE-ALDH7A1 individuals and neonatal controls were up to ~50 µmol/L and ~3 µmol/L, respectively [3]. Although DBS concentrations may differ slightly due to intracellular accumulation in red blood cells, the values we obtained remain substantially higher.

Nevertheless, our interpretation relies on percentile-based thresholds, ensuring robust classification despite the absence of absolute quantification. We recommend that any positive screening result should be confirmed using complementary biochemical assays and ALDH7A1 gene sequencing.

In this study, the duration of sample storage ranged from 9 months to 5 years. Although we demonstrated acceptable stability for the three biomarkers (CV < 21%) over a 6-month period at room temperature in three patients with PDE-ALDH7A1, we cannot rule out the possibility of biomarker degradation between the neonatal period and several months or years later. Prospective studies that assess these biomarkers both in the first days of life and at later time points will be essential to more accurately evaluate their long-term stability on DBS.

Further data from larger sample sizes, prospective DBS samples and unusual late-onset patients will be essential to refine cutoff thresholds and to confirm the good sensitivity and positive predictive value of this approach in NBS programs. It is also important to consider potential false positives arising from unknown or reported ultra-rare conditions such as molybdenum cofactor deficiency, isolated sulfite oxidase deficiency and DHTKD1 deficiency, in which α-AASA and oxo-PIP can also be found elevated [32,36].

Also of note is that 2-OPP measured in DBS may also serve as a useful and minimally invasive tool for biochemical diagnosis to confirm the pathogenicity of genetic variants, or as a valuable indicator for clinical outcome and therapy compliance monitoring of PDE-ALDH7A1 patients undergoing “triple therapy” (i.e., B6, low lysin and high arginine). Furthermore, 2-OPP in DBS or other biological matrix could potentially replace the current use of the less stable biomarkers P6C and α- AASA measured in blood plasma or urine.

We conclude that 2-OPP-based screening preferably combined with PIP or oxo-PIP should be considered for integration into NBS programs using FIA-MS/MS assays, with ongoing validation studies needed to confirm and optimize the approach.

## Figures and Tables

**Table 1:** Precision and reproducibility of the assay for 2-OPP, oxo-PIP and PIP. CV: Coefficient of variation, L: low level, M: medium level, H: high level.

		Intra-Day Variation (Precision)			Inter-Day Variation (Reproducibility)		
	[µmol/L]	Mean	SD	CV (%)	Mean	SD	CV (%)
2-OPP	0.25 (L)	0.35	0.04	11.7	0.34	0.04	10.3
	1 (M)	1.13	0.10	8.4	1.13	0.13	11.9
	5 (H)	5.76	0.34	5.9	5.6	0.51	9.1
Oxo-PIP	0.5 (L)	0.35	0.01	3.8	0.37	0.04	9.2
	2 (M)	0.9	0.11	11.7	1.03	0.18	17.9
	10 (H)	4.09	0.24	5.8	4.38	0.59	13.4
PIP	22.5 (L)	51.3	1.73	3.4	46	6.65	14.5
	112.5 (M)	143	17.1	11.9	156	9.93	6.4
	225 (H)	284	22.9	8.1	507	52.0	10.2

**Table 2:** Genotype, clinical and biochemical findings of the PDE-ALDH7A1 patients. The corresponding calculated 99.9th percentiles of relative concentrations for 2-OPP, oxo-PIP and PIP in DBS are 0.48, 1.13 and 266.3 µmol/L, respectively. P: Pathogenic, LP: likely pathogenic. * PLP instead of B6.

Patient	Genotype	Sex	Age at 1st Seizure	Age at Pyridoxine	2-OPP (µmol/L) P99.5th: 0.31 P99.9th: 0.48	Oxo-PIP (µmol/L) P99.5th: 0.84 P99.9th: 1.13	PIP (µmol/L) P99.5th: 201 P99.9th: 266	Age of DBS at Time of Testing
1	c.834G>A (p.Val278 =) (P/LP); c.1279G>C (p.Glu427Gln) (P/LP)	F	5 days	28 days	1.90	0.90	126	2.5 years
2	c.902A>T (p.Asn301Ile) (P/LP), c.1279G>C (p.Glu427Gln) (P/LP)	M	5 days	16 days	0.45	0.63	125	4 years
3	c.902A>T (p.Asn301Ile) (P/LP); c.1279G>C (p.Glu427Gln) (P/LP)	F	No seizure	0 day	0.82	1.35	147	3 years
4	c.690A-1095_716 delinsG; r.690_787del (exon 9) (LP); c.612–502 G>C; r.612-541_506ins LP)	M	No seizure	0 day	0.71	0.85	148	3 years
5	c.1061A >G (p.Tyr 354 Lys) (P); c.1061A>G (p.Tyr 354 Lys) (P)	M	1 day	2 days	1.43	0.49	124	3 years
6	c.1588del (p.Leu530Phefs*21) (LP); c.1588del (p.Leu530Phefs*21) (LP)	M	2 days	2 days	2.85	1.19	122	9 months
7	c.1279G>C (p.Glu427Gln) (P/LP); c.1279G>C (p.Glu427Gln) (P/LP)	M	H 1	1 day *	1.13	1.46	147	5 years
8	c.916T>A (p.Phe306Ile) (LP); c.1279G>C (p.Glu427Gln) (P/LP)	M	5 days	6 days	3.57	0.97	125	3.5 years
9	c.650 + 260_695 + 950del (P); c.774–1095_801del (LP)	F	3 days	6 days	7.90	1.81	158	23 months

**Table 3:** Performances of PDE-ALDH7A1 biomarkers measured alone or combined together. Data from 9 positive cases and 9393 negative controls. CI: 95% confidence intervals.

Biomarker 1 and Threshold Used in Percentiles	Biomarker 2 and Threshold Used in Percentiles	Sensitivity	Specificity	Positive Predictive Value
2-OPP (P99.9th)	N/A	88.9% (CI:56.5–99.4)	99.98% (CI:99.9–100)	80.0%
Oxo-PIP (P99.5th)	N/A	77.8% (CI:45.26–96.05)	99.6% (CI:99.42–99.69)	14.9%
2-OPP (P99.5th)	N/A	100% (CI:70.1–100)	99.6% (CI:99.5–99.7)	18.4%
2-OPP (P99.89th)	Oxo-PIP (P85.0th)	100% (CI:70.1–100)	100% (CI:99.96–100)	100%
2-OPP (P99.89th)	PIP (P85.0th)	100% (CI:70.1–100.0)	100% (CI:99.96–100)	100%
2-OPP (P99.5th)	Oxo-PIP (P85.0th)	100% (CI:70.1–100)	99.9% (CI:99.8–99.9)	45.0%
2-OPP (P99.5th)	PIP (P85.0th)	100% (CI:70.1–100)	99.9% (CI:99.86–99.97)	60.0%

## Data Availability

The data that support the findings of this study are available from the corresponding author upon reasonable request.

## References

[1] Basura G.J., Hagland S.P., Wiltse A.M., Gospe S.M. (2009). Clinical features and the management of pyridoxine-dependent and pyridoxine-responsive seizures: Review of 63 North American cases submitted to a patient registry. Eur. J. Pediatr..

[2] Mills P.B., Struys E., Jakobs C., Plecko B., Baxter P., Baumgartner M., Willemsen M.A., Omran H., Tacke U., Uhlenberg B. (2006). Mutations in antiquitin in individuals with pyridoxine-dependent seizures. Nat. Med..

[3] Plecko B., Paul K., Paschke E., Stoeckler-Ipsiroglu S., Struys E., Jakobs C., Hartmann H., Luecke T., di Capua M., Korenke C. (2007). Biochemical and molecular characterization of 18 patients with pyridoxine-dependent epilepsy and mutations of the antiquitin (ALDH7A1) gene. Hum. Mutat..

[4] Bok L.A., Struys E., Willemsen M.A., Been J.V., Jakobs C. (2007). Pyridoxine-dependent seizures in Dutch patients: Diagnosis by elevated urinary alpha-aminoadipic semialdehyde levels. Arch. Dis. Child..

[5] Struys E.A., Bok L.A., Emal D., Houterman S., Willemsen M.A., Jakobs C. (2012). The measurement of urinary Delta(1)-piperideine-6-carboxylate, the alter ego of alpha-aminoadipic semialdehyde, in Antiquitin deficiency. J. Inherit. Metab. Dis..

[6] Sadilkova K., Gospe S.M., Hahn S.H. (2009). Simultaneous determination of alpha-aminoadipic semialdehyde, piperideine-6-carboxylate and pipecolic acid by LC-MS/MS for pyridoxine-dependent seizures and folinic acid-responsive seizures. J. Neurosci. Methods.

[7] Plecko B., Hikel C., Korenke G.C., Schmitt B., Baumgartner M., Baumeister F., Jakobs C., Struys E., Erwa W., Stockler-Ipsiroglu S. (2005). Pipecolic acid as a diagnostic marker of pyridoxine-dependent epilepsy. Neuropediatrics.

[8] Plecko B., Stockler-Ipsiroglu S., Paschke E., Erwa W., Struys E.A., Jakobs C. (2000). Pipecolic acid elevation in plasma and cerebrospinal fluid of two patients with pyridoxine-dependent epilepsy. Ann. Neurol..

[9] Kawasaki H., Hori T., Nakajima M., Takeshita K. (1988). Plasma levels of pipecolic acid in patients with chronic liver disease. Hepatology.

[10] Peduto A., Baumgartner M.R., Verhoeven N.M., Rabier D., Spada M., Nassogne M.C., Poll-The B.T., Bonetti G., Jakobs C., Saudubray J.M. (2004). Hyperpipecolic acidaemia: A diagnostic tool for peroxisomal disorders. Mol. Genet. Metab..

[11] Houten S.M., Te Brinke H., Denis S., Ruiter J.P., Knegt A.C., de Klerk J.B., Augoustides-Savvopoulou P., Haberle J., Baumgartner M.R., Coskun T. (2013). Genetic basis of hyperlysinemia. Orphanet J. Rare Dis..

[12] Bok L.A., Halbertsma F.J., Houterman S., Wevers R.A., Vreeswijk C., Jakobs C., Struys E., Van Der Hoeven J.H., Sival D.A., Willemsen M.A. (2012). Long-term outcome in pyridoxine-dependent epilepsy. Dev. Med. Child Neurol..

[13] Coughlin C.R., Tseng L.A., Bok L.A., Hartmann H., Footitt E., Striano P., Tabarki B.M., Lunsing R.J., Stockler-Ipsiroglu S., Gordon S. (2022). Association Between Lysine Reduction Therapies and Cognitive Outcomes in Patients With Pyridoxine-Dependent Epilepsy. Neurology.

[14] Coughlin C.R., van Karnebeek C.D., Al-Hertani W., Shuen A.Y., Jaggumantri S., Jack R.M., Gaughan S., Burns C., Mirsky D.M., Gallagher R.C. (2015). Triple therapy with pyridoxine, arginine supplementation and dietary lysine restriction in pyridoxine-dependent epilepsy: Neurodevelopmental outcome. Mol. Genet. Metab..

[15] Mahajnah M., Corderio D., Austin V., Herd S., Mutch C., Carter M., Struys E., Mercimek-Mahmutoglu S. (2016). A Prospective Case Study of the Safety and Efficacy of Lysine-Restricted Diet and Arginine Supplementation Therapy in a Patient With Pyridoxine-Dependent Epilepsy Caused by Mutations in ALDH7A1. Pediatr. Neurol..

[16] Mercimek-Mahmutoglu S., Cordeiro D., Cruz V., Hyland K., Struys E.A., Kyriakopoulou L., Mamak E. (2014). Novel therapy for pyridoxine dependent epilepsy due to ALDH7A1 genetic defect: L-arginine supplementation alternative to lysine-restricted diet. Eur. J. Paediatr. Neurol..

[17] van Karnebeek C.D., Hartmann H., Jaggumantri S., Bok L.A., Cheng B., Connolly M., Coughlin C.R., Das A.M., Gospe S.M., Jakobs C. (2012). Lysine restricted diet for pyridoxine-dependent epilepsy: First evidence and future trials. Mol. Genet. Metab..

[18] Yuzyuk T., Thomas A., Viau K., Liu A., De Biase I., Botto L.D., Pasquali M., Longo N. (2016). Effect of dietary lysine restriction and arginine supplementation in two patients with pyridoxine-dependent epilepsy. Mol. Genet. Metab..

[19] Al Teneiji A., Bruun T.U., Cordeiro D., Patel J., Inbar-Feigenberg M., Weiss S., Struys E., Mercimek-Mahmutoglu S. (2017). Phenotype, biochemical features, genotype and treatment outcome of pyridoxine-dependent epilepsy. Metab. Brain Dis..

[20] Falsaperla R., Vari M.S., Toldo I., Murgia A., Sartori S., Vecchi M., Suppiej A., Burlina A., Mastrangelo M., Leuzzi V. (2018). Pyridoxine-dependent epilepsies: An observational study on clinical, diagnostic, therapeutic and prognostic features in a pediatric cohort. Metab. Brain Dis..

[21] van Karnebeek C.D., Tiebout S.A., Niermeijer J., Poll-The B.T., Ghani A., Coughlin C.R., Van Hove J.L., Richter J.W., Christen H.J., Gallagher R. (2016). Pyridoxine-Dependent Epilepsy: An Expanding Clinical Spectrum. Pediatr. Neurol..

[22] Mills P.B., Footitt E.J., Mills K.A., Tuschl K., Aylett S., Varadkar S., Hemingway C., Marlow N., Rennie J., Baxter P. (2010). Genotypic and phenotypic spectrum of pyridoxine-dependent epilepsy (ALDH7A1 deficiency). Brain.

[23] Mercimek-Mahmutoglu S., Horvath G.A., Coulter-Mackie M., Nelson T., Waters P.J., Sargent M., Struys E., Jakobs C., Stockler-Ipsiroglu S., Connolly M.B. (2012). Profound neonatal hypoglycemia and lactic acidosis caused by pyridoxine-dependent epilepsy. Pediatrics.

[24] Dowa Y., Shiihara T., Akiyama T., Hasegawa K., Inoue F., Watanabe M. (2020). A case of pyridoxine-dependent epilepsy with novel ALDH7A1 mutations. Oxf. Med. Case Rep..

[25] Coughlin C.R., Tseng L.A., van Karnebeek C.D.M. (2022). A case for newborn screening for pyridoxine-dependent epilepsy. Mol. Case Stud..

[26] Hess-Homeier D.L., Cunniff C., Grinspan Z.M. (2019). Priorities for Newborn Screening of Genetic Epilepsy. Pediatr. Neurol..

[27] Wempe M.F., Kumar A., Kumar V., Choi Y.J., Swanson M.A., Friederich M.W., Hyland K., Yue W.W., Van Hove J.L.K., Coughlin C.R. (2019). Identification of a novel biomarker for pyridoxine-dependent epilepsy: Implications for newborn screening. J. Inherit. Metab. Dis..

[28] Jung S., Tran N.T., Gospe S.M., Hahn S.H. (2013). Preliminary investigation of the use of newborn dried blood spots for screening pyridoxine-dependent epilepsy by LC-MS/MS. Mol. Genet. Metab..

[29] Mathew E.M., Moorkoth S., Lewis L., Rao P. (2018). Biomarker Profiling for Pyridoxine Dependent Epilepsy in Dried Blood Spots by HILIC-ESI-MS. Int. J. Anal. Chem..

[30] Engelke U.F., van Outersterp R.E., Merx J., van Geenen F.A., van Rooij A., Berden G., Huigen M.C., Kluijtmans L.A., Peters T.M., Al-Shekaili H.H. (2021). Untargeted metabolomics and infrared ion spectroscopy identify biomarkers for pyridoxine-dependent epilepsy. J. Clin. Investig..

[31] Damiano R., Della Bona M., Procopio E., Guerrini R., Bettiol A., la Marca G. (2025). Inclusion of pyridoxine dependent epilepsy in expanded newborn screening programs by tandem mass spectrometry: Set up of first and second tier tests. Clin. Chem. Lab. Med..

[32] Pauly K., Woontner M., Abdenur J.E., Chaudhari B.P., Gosselin R., Kripps K.A., Thomas J.A., Wempe M.F., Gospe S.M., Coughlin C.R. (2025). Feasibility of newborn screening for pyridoxine-dependent epilepsy. Mol. Genet. Metab..

[33] Dewulf J.P., Chevalier N., Marie S., Veiga-da-Cunha M. (2023). DBS are suitable for 1,5-anhydroglucitol monitoring in GSD1b and G6PC3-deficient patients taking SGLT2 inhibitors to treat neutropenia. Mol. Genet. Metab..

[34] Trinh M.U., Blake J., Harrison J.R., Gerace R., Ranieri E., Fletcher J.M., Johnson D.W. (2003). Quantification of glutamine in dried blood spots and plasma by tandem mass spectrometry for the biochemical diagnosis and monitoring of ornithine transcarbamylase deficiency. Clin. Chem..

[35] Li X., Ding Y., Liu Y., Ma Y., Song J., Wang Q., Yang Y. (2015). Five Chinese patients with 5-oxoprolinuria due to glutathione synthetase and 5-oxoprolinase deficiencies. Brain Dev..

[36] Mills P.B., Footitt E.J., Ceyhan S., Waters P.J., Jakobs C., Clayton P.T., Struys E.A. (2012). Urinary AASA excretion is elevated in patients with molybdenum cofactor deficiency and isolated sulphite oxidase deficiency. J. Inherit. Metab. Dis..

